# Risk Prediction and Assessment of Intervention, Re-education and Reintegration of Juvenile Offenders: Development and Psychometric Properties of the PREVI-A

**DOI:** 10.3389/fpsyg.2022.896573

**Published:** 2022-06-20

**Authors:** José Luis Graña Gómez, Román Ronzón-Tirado, José Manuel Andreu Rodríguez, María Elena de la Peña Fernández

**Affiliations:** ^1^Department of Personality, Assessment and Clinical Psychology, Faculty of Psychology, Complutense University of Madrid, Madrid, Spain; ^2^Department of Biological and Health Psychology, Faculty of Psychology, Autonomous University of Madrid, Madrid, Spain

**Keywords:** risk assessment, juvenile offender, recidivism, case management, delinquency

## Abstract

This paper proposes and analyzes the psychometric properties of the PREVI-A scale (*Predicción del Riesgo y Valoración de la Intervención en la ARRMI*—-Risk Prediction and Intervention Assessment in the ARRMI). It describes the process of item development, the factorial structure of the scale, reliability, evidence of validity and diagnostic performance with regard to recidivism risk in juvenile offenders. The sample was made up of 212 juvenile offenders held at detention centers run by the Madrid Agency for Reeducation and Reintegration of Juvenile Offenders, a regional government body. Statistical analyses were used to corroborate the theoretical factorial structure of the PREVI-A, which consists of six risk/protection dimensions (64 items) based on the Risk-Needs-Responsivity Model, and to obtain empirical support for the reliability and validity of PREVI-A as a tool to assess the risk of recidivism by juvenile offenders in Spain.

## Introduction

A growing number of researchers have become interested in the analysis of violent behavior, patterns of recidivism and desistance in young offenders over the last two decades, (Andrews and Bonta, 2010a; Andreu-Rodríguez et al., 2015; Horcajo-Gil et al., 2019; Borum et al., 2020). Their work reveals the need for standard standardized tools to assess not only specific youth recidivism risks and protective factors, but also the personal and environmental needs that could be addressed in juvenile detention to enhance the desistance process (Echeburúa et al., 2011; Baglivio and Jackowski, 2013).

At the design level, numerous instruments and strategies developed to understand criminal behavior, which can be summarized in four generations according to the progress made in terms of measurement reliability, diagnostic capacity and intervention (Andrews and Bonta, 2003). The first, and least reliable, approach was the assessment of recidivism risk of based on the clinical judgment of professionals. This stage was characterized by a lack of objectivity affecting measurement and by replication problems. The second generation was based on actuarial assessment using weighted statistical predictors and significance levels for recidivism, but it was largely bereft of theoretical foundations. This was followed by a third generation which sought to combine risk and protection factors to achieve a theory-based static assessment of risk vs. non-risk, and finally, by the present fourth generation of instruments based on the insight that risk needs to be assessed as a continuous and dynamic process in order to link up evaluation results with case management and the actual treatment of minors. This approach assumes that ongoing re-evaluation of juvenile offenders over the course of the rehabilitation process is key to successful risk assessment with the ultimate goal of leveraging each youth's individual strengths, learning styles, and coping resources to bolster desistance from delinquency (Bonta, 2002; Barnes et al., 2016; Campbell et al., 2018).

The Risk-Needs-Responsivity Model (RNR) proposed by Andrews and Bonta (2010b) based on this fourth generation approach is currently widely accepted theoretical explanations applied to the design of effective treatment measures for violent and delinquent behavior (Andrés-Pueyo and Echeburúa, 2010; Horcajo-Gil et al., 2019). The RNR model assumes that criminal behavior is a learned behavior acquired in a specific social context involving the interaction of both static (history of antisocial behavior) and dynamic risk factors (impulsivity and antisocial tendencies, belief systems, drug use, and peer group influence, respectively) related to higher or lower levels of risk and modifiability of criminal behavior (Andrews et al., 1990). The distinction between static and dynamic factors relays on whether the risk factor can be modified or not. Historical precursors such as the seriousness of the committed crimes, time immersed in the criminal behavior, evidence of antisocial behavior before age 14, or the unresolved open court cases are useful for predicting the risk of recidivism and evaluating the minor's intervention needs but are not amenable to change to reduce the risk (McGrath and Thompson, 2012). While dynamic risk factors refer to the conditions that are currently related to offending and are potentially modifiable (Andrews et al., 1990) such as lack of adequate conflict resolution strategy/negotiation skills, having attributional biases, having low frustration tolerance, or the lack of strategies to withstand peer pressure.

The RNR model thus seeks to explain individual differences in criminal behavior based on influences or reinforcements from the broader cultural, social, familial, interpersonal and individual context. In this light, assessment involves the classification of risk/protective factors into eight major dimensions (Andrews and Bonta, 2010b), four of them dubbed “the big four” for their strong predictive value (history of antisocial behavior [static factor], antisocial personality pattern, antisocial cognition, and antisocial peer affiliations) and “the moderate four” (family/marital circumstances, school/work, leisure/recreation; and substance abuse), given their moderate relationship with criminal recidivism (Assink et al., 2015).

The RNR Model (Andrews and Bonta, 2010b) has been widely accepted in the United States and is used as the benchmark for assessments of juvenile offenders in the Washington State Juvenile Court Assessment (WSJCA; Barnoski, 2004) and the Youth Level of Service and Case Management Inventory (YLS/CMI; Hoge, 2001). Both of these instruments have demonstrated predictive validity for delinquent behavior in juvenile offenders with mean AUC effects ranging from 0.579 to 0.67 (Schmidt et al., 2005; Olver et al., 2009). Despite abundant evidence of test validity in the White Anglo-Saxon population, however, recent research suggests differential prediction of delinquent behavior in different cultural and ethnic groups (Onifade et al., 2008; Barnes et al., 2016), tending to a biased overstatement of risk in certain cultures and ethnicities. For instance, Campbell et al. (2018), found a significant interaction between race, gender, and risk score of the YLS when predicting recidivism, differing significantly for black youth when compared to white youth. Moreover, McCafferty (2018) found the risk of recidivism in 2,600 juvenile offenders was overestimated for Black youth due to methodological factors rather than empirical realities. Overall risk assessments seem to overestimate risk for certain juveniles, especially those how to belong to minority groups, who have a history of being marginalized, or who belong to a different culture to the one in which the instrument was created (Chenane et al., 2015; Henderson et al., 2015). From the standpoint of continuous assessment/rating, the poor cultural adaptation of the test to other target samples might limit not only the predictive capacity of the scales but also the opportunity to consider the influence of cultural and ethnic factors on the treatment and re-education of juvenile offenders and their eventual desistance from delinquency (Mallett and Stoddard-Dare, 2010; Campbell et al., 2018).

It is evident that culturally adapted instruments are urgently needed to improve existing procedures to assess the risk of recidivism and track the evolution of young offenders throughout their developmental cycle. Such tools would enable the courts to impose more effective measures based on minors' actual needs in a specific cultural context, and would help researchers and professionals understand changes in offenders' behavior and fine tune educational and therapeutic intervention programs. In the Autonomous Community of Madrid (Spain), the regional Agency for the Re-education and Reintegration of Young Offenders (*Agencia para la Reeducación y Reinserción del Menor Infractor* or ARRMI) is tasked with enforcing court mandated measures in juvenile delinquency cases. ARRMI's daily work has provided the agency with ample opportunity to observe that the assessment measures generally used in Spain are not sufficiently sensitive to assess recidivism risk or behavioral changes in juvenile offenders held in detention. Moreover, certain items included in prior tolls such as drug use, were not meant to be assessed during detention time, minors had no access to drugs while they were in detention., while the available (third generation) measures take a static approach to risk assessment, making it difficult to track the evolution of risk over time. In order to address these shortcomings, ARRMI decided to develop a proper assessment tool for the Spanish context and culture capable of addressing recidivism risk as a dynamic process from the perspective of RNR model. PREVI-A is meant to be used as a tool for assessing the risk of recidivism but mostly for the follow up of the minors and change on risk over time, adjusting the planning and intervention for each minor according to their scores. Some of the more specific questions are included in the social-interpersonal integration or the socio-familiar integration (e.g., Lacks strategies to withstand peer pressure; little or no presence of structured activities; Refuses intervention or does not know how to access social resources or socio community resources; Reversal of parent/child roles). These areas when scored as “high risk” are set out as intervention objectives for which specific strategies are implemented by the psychologist and social workers from the ARMMI for each minor. As time passes and the objectives set in the initial risk assessments are addressed, ARMMI psychologists and social workers can evaluate the decrease in risk, this task could not be done with the previously existing instruments. This was the origin of the PREVI-A tool (Risk Prediction and Assessment of Intervention in ARRMI).

### PREVI-A: Risk Prediction and Assessment of Intervention in ARRMI

PREVI-A was built in response to the need for a valid tool to assess and manage the risk of recidivism among the inmates and open-regime juvenile offenders at the juvenile correction centers maintained by the Autonomous Community of Madrid, known as *Ciudades Escuela de los Muchachos* (CEMU). It is the result of more than 4 years' work by over 200 professionals and experts in the field of custodial and non-custodial enforcement from ARRMI and the Complutense University of Madrid, Spain [masked for peer-review process] involved in the design of the scale, and the classification and assessment of each of the items. PREVI-A is made up of 64 items grouped in six dimensions comprising (1) Legal Situation (aspects related to the crime itself, and the associated judicial response and institutional trajectory); (2) Context and Intervention (assimilation of coexistence rules and adaptation to the environment, responsibility and commitment to change); (3) School, Occupational Training and Work (situation of the young offender with respect to occupational training and work); (4) Personal Development (cognitive, emotional and behavioral aspects, psychopathology and substance use); (5) Socio-Familial Integration (parent-child relationships, relationships between parents, family model); and (6) Social/Interpersonal Integration (relationship groups, leisure and free time, social network). This study proposes and validates the PREVI-A scale, describing the process of item development, factorial structure, reliability, evidence of validity and diagnostic performance regarding the risk of criminal recidivism.

## Method

### Participants

The study involved 212 juvenile offenders attending CEMU correction centers run by the Madrid regional Agency for the Re-education and Reintegration of Young Offenders (ARRMI) in the Autonomous Community of Madrid, Spain. The mean age of the juvenile offenders taking part was 16.61 years (*SD* = 1.06, range 14–18), while 13.7% (*n* = 29) were female and 86.3% (*n* =183) were male. In terms of nationality, 43.9% (*n* = 93) were Spanish, 27.8% (*n* = 59) were Latin American, 15.1% (*n* = 32) were of North African origin, 9.9% (*n* = 28) were from the rest of Europe and 3.3% (*n* = 7) were from other countries. Meanwhile, 17.4% (*n* = 37) of the participants perceived their socioeconomic level as very low, 43.8% (*n* = 93) as low, 35.8% (*n* = 76) as medium and 3.3% (*n* = 7) as high. Finally, 44.81% (*n* = 95) of these youths were serving probationary sentences, 44.81% (*n* = 95) were in semi-open detention and 10.37% (*n* = 22) were in full detention. Around 63% of the sample had committed crimes against property, 7.2% crimes against freedom of a person; 7.2% homicide; 7% injuries and around 15% other crimes such as disturbances of public order or security crimes.

### Instruments

#### Risk Prediction and Assessment of Intervention in ARRMI (PREVI-A)

PREVI-A is a hetero-applied scale (every 3 months) for the assessment and management of the risk of delinquent behavior among juvenile offenders based on the Risk-Needs-Responsivity Model proposed by Andrews and Bonta (2010a,b). PREVI-A is designed to be applied by juvenile delinquency professionals based on information obtained from interviews held with young offenders, parents and other professionals, supported by data obtained from other sources (e.g., court records, academic records, and observation of the child's behavior). The scale consists of 64 Likert-type items covering six dimensions: Legal Situation (seven items), Context and Intervention (12 items), School, Occupational Training and Work (seven items), Personal Development (21 items), Socio-Familial Integration (eight items) and Social/Interpersonal Integration (nine items). The response format of the items allows discrimination of the difference in frequency/intensity of risk with respect to the assessed behavior, where 0 corresponds to the absence of risk and 3 to maximum risk (0 = “*never or almost never/no risk*,” 1 = “*sometimes/low risk*,” 2 = “*frequent/medium risk*,” 3 = “*always or almost always/high risk*”). At the end of each dimension, a section is included to allow description of key aspects so as to ensure that all elements required for the purposes of risk assessment and the formulation of specific intervention objectives are included. The risk score obtained is the sum of the total items by dimension.

#### Youth Level of Service/Case Management Inventory (YLS/CMI)

Spanish adaptation (Inventario de Gestión e Intervención para Jóvenes; IGI-J) proposed by Garrido et al. (2004). This inventory was used to examine the convergent validity of the PREVIA. The YLS/CMI (Hoge and Andrews, 2002) is a hetero-applied questionnaire for the screening of risk and protective factors relevant to decision-making on the level of intervention and supervision of juvenile offenders. It is formed by 42 items that assess the presence/absence of risk/protective factors distributed in eight dimensions: criminal history (e.g., “*non-compliance and violations of judicial measures*”), educational patterns (e.g., “*inadequate supervision*”), education/employment (e.g., “*disruptive behavior in class/work*”), peer group (e.g., “*some friend of his is a delinquent*”), leisure/fun (e.g., “*does not show personal interests*”), drug use (e.g., “*occasional drug use*”), personality/behavior (e.g., “*fits of anger*”), and attitudes/beliefs (e.g., “*actively refuses help*”). Total scores equal to or lower than 8 indicate low levels of risk, while scores equal to or higher than 22 are equivalent to high levels of risk. In the present study, the questionnaire obtained adequate internal consistency indices for the eight dimensions ranging from α = 0.78 (0.72–0.81) to α = 0.93 (0.91–0.94).

### Procedure

First, the research team had a reunion with the directors of the CEMUs and discussed ARMMI's needs, they designed the research project based on RNR model. CEMUs directors meet with the psychologist and social workers of each center and gather potential items and factors based on their experience on the assessment and management of recidivism over the years of their work at the Young Offenders Agency. Then, the research team along with 14 directors of ARRMI centers designed the bank of 110 items to assess the risk of violent behavior based on the Risk-Needs-Responsivity Model proposed by Andrews and Bonta (2010a,b) and the suggestions made by the psychologist and social workers of the CEMUs. Next, 134 ARRMI professionals from both custodial and non-custodial settings (technical and social educators, social workers and psychologists) were asked to score the quality and classification of each of the 110 items making up the six theoretical risk assessment dimensions on a scale from 0 to 10. The items, as reworded in the case of any inadequately formulated statements were then screened based on the score obtained and the dispersion of the results, resulting in a total of 64 items. The inter-judge agreement index for the assessment of the items and classification was almost perfect, with values equivalent to 74%−86%. Throughout the year a total of 212 minors were evaluated (first wave) and the 54.7% (*n* = 116) of them were evaluated for a second time after 3 months of the first assessment (second wave) to address their evolution scores. The PREVI-A questionnaire was completed by ARRMI professionals as part of their work of monitoring, assessment and control over judicial measures applied to juvenile offenders. This task took around 60 min.

### Data Analyses

The descriptive statistics and internal consistency for the instruments used in the study were first analyzed using *Cronbach*'*s Alpha* and the *Omega* coefficient. A confirmatory factor analysis (CFA) was then estimated for PREVI-A using the Weighted Least Squares Mean and Variance Adjusted (WLMSV) estimator in view of its robustness in the case of ordinal scales like the PREVI-A, and because it offers excellent statistical guarantees since the weighting matrix is estimated based on the asymptotic variances and covariances of polychoric correlations. This estimator assumes a latent normal distribution underlying each categorical variable observed, and non-normality and non-independence of observations (Byrne, 2012; Li, 2016). The indexes and values used to examine the model's fit included the Comparative Fit Index (CFI; Acceptable Fit = CFI ≥ 0.9), Tucker-Lewis index (TLI; Acceptable Fit = TLI ≥ 0.9) and the Root Mean Square Error of Approximation (RMSEA; Acceptable Fit. RMSEA ≤ 0.08; Jöreskog, 2001), while the criterion for keeping/removing items (scale reduction) and confirming the final scale structure through the CFA was Factor loadings >0.4; *R*^2^ > 0.02 (Netemeyer et al., 2003; Hooper et al., 2008). Following corroboration of the factorial structure of PREVI-A, the internal consistency of the 6 dimensions of the scale was tested using the Cronbach's Alpha (α) and Omega (ω) coefficients. Internal consistency coefficients ≥0.70 were considered “acceptable” indicators, ≥0.80 “good,” and ≥0.90 “excellent” (Taber, 2018).

The test-retest reliability of PREVI-A was then examined applying the Spearman correlation coefficient (*rs*) in two applications of the instrument after a period of 3 months. Scores of *rs* > 0.70 were considered indicators of consistency and temporal measurement (Polit, 2014). After analyzing the reliability indicators of PREVI-A, evidence of the scale's concurrent validity was checked by comparing the Spearman correlations of the six PREVI-A dimensions with the YLS/CMI eight dimensions. Meanwhile, the evidence for the predictive validity of the scale with regard to the risk of criminal recidivism was checked using logistic regression models. To analyze predictive validity, cases were classified in terms of (a) recidivism, defined as a situation in which a minor is currently serving a sentence for one or more offenses, has a pending court appearance for another offense, and has a prior criminal record); or (b) non-recidivism, defined as a situation in which a minor is currently serving a sentence for one or more offenses, has no pending court appearances for any other offenses, and has no prior criminal record). Finally, the diagnostic performance of PREVI-A was analyzed by means of a Receiver Operating Characteristics (*ROC*) curve on recidivism or non-recidivism of juvenile offenders with values of the area under the curve >0.70 considered indicators of good classification quality (Rice and Harris, 2005). These analyses were conducted using the *M-plus* version 7 statistical package and the *ROCit* package version 2.1.1 of n R statistics.

## Results

### Confirmatory Factor Analysis

The confirmatory factor analysis was configured on the six theoretical dimensions proposed. The results showed a good model fit with scores of CFI = 0.927; TLI = 0.924; RMSEA = 0.059 [0.057 0.064]. Meanwhile, analysis of the factor weights and *R*^2^ values of the 64 items showed that all of them were satisfactory in both cases.

### Internal Consistency and Reliability

The internal consistency analysis of PREVI-A found “good” to “excellent” indices of internal consistency for each of the dimensions with α = 0.70–0.95 and ω = 0.71–0.96 (Table 1 [Spanish version]; Table 2 [English version]). The test-retest reliability of the PREVI-A, calculated using Spearman's correlation coefficient in two applications after a period of 3 months, was “good,” corroborating the temporal stability of the scale with *rs* = 0.71 [0.70 0.73] (*p* < 0.001).

**Table 1:** Scale Items and Psychometric Properties for PREVI-A dimensions (español).

	**α**	**ω**	**Factor loading**	***M***	***SD***
**Área Jurídico legal. (Legal situation)**	0.70	0.71			
1. Gravedad de los delitos cometidos, entendida como presencia de violencia			0.546^***^	1.21	0.81
2. Incumplimiento / quebrantamientos en medida actual o medidas anteriores			0.636^***^	0.89	1.19
3. Versatilidad delictiva			0.560^***^	0.57	0.75
4. Tiempo que lleva inmerso en las conductas delictivas			0.701^***^	1.15	1.04
5. Expedientes judiciales abiertos sin resolver			0.582^***^	0.33	0.58
6. Existencia de conductas antisociales significativas en edad penal que no hayan supuesto la apertura de expediente judicial e imposición de medida judicial			0.618^***^	0.90	1.06
7. Constancia de conductas antisociales antes de los 14 años			0.527^***^	0.53	0.93
**Área de contexto e intervención. (Context and intervention)**	0.91	0.91			
1. Presencia/ausencia de episodios de comunicación agresiva o violenta con iguales			0.762^***^	0.91	0.88
2. Relación con figuras de autoridad. Asunción/oposicionismo			0.799^***^	1.20	1.02
3. Daña espacios y/o material			0.600^***^	0.49	0.78
4. Coacciona o coarta a sus compañeros o iguales para imponer criterios o acciones			0.650^***^	0.50	0.71
5. Incumplimiento de la normativa establecida			0.912^***^	1.28	1.02
6. Presenta dificultades para asunción/interiorización de las normas			0.909^***^	1.30	1.08
7. Incumplimiento de horarios			0.770^***^	1.11	1.13
8. Presenta conductas de riesgo en ámbitos esenciales de salud			0.753^***^	1.04	1.07
9. Presenta dificultades para la asunción y comprensión del delito como daños para los demás			0.859^***^	1.50	1.12
10. Presenta dificultades para la responsabilización ante el delito			0.853^***^	1.48	1.11
11. Presenta dificultades para la colaboración en las intervenciones profesionales			0.768^***^	1.19	0.92
12. Presenta dificultades para el cumplimiento de obligaciones establecidas en la medida judicial			0.755^***^	1.01	0.95
**Área escolar, formativa, prelaboral, laboral. (School, occupational training and work)**	0.90	0.90			
1. Problemas con los profesores/superiores			0.823^***^	0.88	0.99
2. Falta de interés por la actividad formativa o laboral			0.919^***^	1.59	1.10
3. Desinterés de los padres por la formación escolar/labora propia del momento evolutivo del menor			0.510^***^	0.98	1.08
4. Problemas para la percepción de beneficio personal a través de la formación/empleo			0.857^***^	1.30	1.05
5. Bajo rendimiento académico/laboral			0.884^***^	1.83	1.07
6. Retraso curricular y/o absentismo/fracaso escolar o laboral			0.819^***^	1.82	1.08
7. Problemas en hábitos de trabajo o desarrollo de las capacidades necesarias para una adecuada competencia en el área formativo laboral			0.915^***^	1.66	0.98
**Área de desarrollo personal (Personal development)**	0.95	0.96			
1. Pensamiento rígido o poco flexible			0.774^***^	1.58	0.96
2. Sesgos atribucionales (suspicacia/hostilidad)			0.798^***^	1.42	1.03
3. Valores antisociales			0.899^***^	1.66	1.03
4. Ausencia de responsabilización y/o falta de remordimientos o culpa sobre las conductas desviadas de la norma			0.846^***^	1.56	1.02
5. Falta de previsión de las consecuencias de la conducta y/o percepción- valoración inadecuada de situaciones de riesgo			0.881^***^	1.74	0.94
6. Falta de reactividad emocional/irascibilidad/irritabilidad			0.784^***^	1.43	1.04
7. Baja empatía. Frialdad emocional			0.806^***^	1.46	0.97
8. Uso frecuente de la mentira			0.608^***^	1.25	0.95
9. Baja tolerancia a la frustración			0.840^***^	1.74	0.94
10. Autoestima inflada o desajustada			0.516^***^	1.08	1.01
11. Impulsividad o bajo autocontrol			0.809^***^	1.56	0.99
12. Desafía a la autoridad/oposicionismo			0.880^***^	1.40	1.02
13. Desinhibición o búsqueda de sensaciones			0.781^***^	1.27	1.02
14. Uso de la violencia/agresividad como recurso presente en el repertorio comportamental del menor			0.838^***^	1.46	1.06
15. Carece de estrategia adecuadas para la resolución de conflictos/habilidades de negociación			0.798^***^	1.69	0.98
16. Comportamientos basados en objetivos inmediatos, materiales y externos			0.874^***^	1.80	1.07
17. Afectación por experiencia traumática			0.468^***^	0.94	1.11
18. Abuso del consumo de drogas			0.875^***^	1.32	1.16
19. El consumo de drogas interfiere de forma importante de su vida/ delitos relacionados con el consumo			0.860^***^	1.13	1.18
20. Amigos con consumo habitual			0.769^***^	1.90	1.09
21. Actitud favorable frente al consumo			0.757^***^	1.38	1.06
**Área de integración socio-familiar-relación familiar. (Socio-familial integration)**	0.85	0.85			
1. Ausencia de normas y límites en el sistema familiar			0.754^***^	1.72	1.09
2. Pautas educativas contradictorias, incoherentes o inconsistentes			0.742^***^	1.75	1.13
3. Comunicación y mensajes negativos en forma continua. Infravaloración del hijo			0.515^***^	0.89	1.07
4. Dificultad o incapacidad para controlar el comportamiento del menor			0.929^***^	2.02	1.07
5. Resistencia activa a las pautas educativas			0.916^***^	1.50	1.15
6. Estilo de vida independiente, ajeno a la vida familiar			0.702^***^	1.38	1.18
7. Experiencia de maltrato familiar (directo o indirecto)			0.488^***^	0.88	1.17
8. Inversión o simetría de roles padres/hijo			0.623^***^	0.95	1.11
**Área de integración social/interpersonal. (Social-interpersonal integration)**	0.89	0.89		1.94	1.09
1. Grupo de relación antisocial			0.881^***^	0.93	1.16
2. Se relaciona con grupo violento organizado			0.500^***^	0.47	0.76
3. Carece de estrategias para soportar la presión de grupo			0.551^***^		
4. Escasa o nula presencia de actividades estructuradas			0.940^***^	2.11	1.08
5. El tiempo de ocio está desocupado y vacío de contenidos			0.950^***^	2.13	1.05
6. Sus intereses y aficiones personales son desadaptativas			0.954^***^	1.70	1.18
7. Se relaciona y se mueve en entornos marginales o de alta conflictividad social			0.796^***^	1.62	1.14
8. Carece de redo social de apoyo			0.630^***^	1.02	1.07
9. Rechaza la intervención o no sabe cómo acceder a recursos sociales o recursos sociocomunitarios			0.806^***^	1.33	1.16

*** *p < 0.001*.

**Table 2:** Scale Items and Psychometric Properties for PREVI-A dimensions (english).

	**α**	**ω**	**Factor loading**	***M***	***SD***
**Legal situation**	0.70	0.71			
1. Seriousness of the committed crimes (the presence of violence).			0.546^***^	1.21	0.81
2. Non-compliance / violations in current or previous measures			0.636^***^	0.89	1.19
3. Criminal versatility			0.560^***^	0.57	0.75
4. Time immersed in criminal behavior			0.701^***^	1.15	1.04
5. Unresolved open court cases			0.582^***^	0.33	0.58
6. Existence of significant antisocial behavior that has not led to the opening of legal proceedings and the imposition of a judicial measure.			0.618^***^	0.90	1.06
7. Evidence of antisocial behavior before age 14			0.527^***^	0.53	0.93
**Context and intervention**	0.91	0.91			
1. Presence/absence of episodes of aggressive or violent communication with peers			0.762^***^	0.91	0.88
2. Relationship with authority figures. Assumption/oppositionism			0.799^***^	1.20	1.02
3. Damages spaces and/or material			0.600^***^	0.49	0.78
4. Coerces peers or equals to impose criteria or actions			0.650^***^	0.50	0.71
5. Non-compliance with established regulations			0.912^***^	1.28	1.02
6. Presents difficulties in the assumption/internalization of norms.			0.909^***^	1.30	1.08
7. Does not comply with schedules			0.770^***^	1.11	1.13
8. Risk behaviors in essential health domains			0.753^***^	1.04	1.07
9. Difficulties in the assumption and understanding of the crime as harm to others.			0.859^***^	1.50	1.12
10. Difficulties for the accountability for the crime			0.853^***^	1.48	1.11
11. Difficulties for collaboration in professional interventions.			0.768^***^	1.19	0.92
12. Difficulties for the fulfillment of obligations established in the judicial measure			0.755^***^	1.01	0.95
**School, occupational training and work**	0.90	0.90			
1. Problems with teachers/superiors			0.823^***^	0.88	0.99
2. Lack of interest in the training or work activity.			0.919^***^	1.59	1.10
3. Lack of parental interest in school/work training appropriate to the child's developmental stage			0.510^***^	0.98	1.08
4. Problems for the perception of personal benefit through training/employment.			0.857^***^	1.30	1.05
5. Low academic/employment performance			0.884^***^	1.83	1.07
6. Curricular delay and/or absenteeism/failure at school or work			0.819^***^	1.82	1.08
7. Problems in work habits or development of the skills necessary for adequate competence in the work training area.			0.915^***^	1.66	0.98
**Personal development**	0.95	0.96			
1. Rigid or inflexible thinking			0.774^***^	1.58	0.96
2. Attributional biases (suspiciousness/hostility)			0.798^***^	1.42	1.03
3. Antisocial values			0.899^***^	1.66	1.03
4. Absence of repsonsabilización and/or lack of remorse or guilt about the deviant behaviors of the norm.			0.846^***^	1.56	1.02
5. Lack of foresight of the consequences of the behavior and/or inadequate perception-assessment of risk situations.			0.881^***^	1.74	0.94
6. Lack of emotional reactivity/irascibility/irritability			0.784^***^	1.43	1.04
7. Low empathy. Emotional coldness			0.806^***^	1.46	0.97
8. Frequent use of lies			0.608^***^	1.25	0.95
9. Low frustration tolerance			0.840^***^	1.74	0.94
10. Inflated or maladjusted self-esteem			0.516^***^	1.08	1.01
11. Impulsivity or low self-control			0.809^***^	1.56	0.99
12. Challenges authority/oppositionism			0.880^***^	1.40	1.02
13. Disinhibition or sensation seeking			0.781^***^	1.27	1.02
14. Use of violence/aggressiveness as a resource present in the child's behavioral repertoire.			0.838^***^	1.46	1.06
15. Lacks adequate conflict resolution strategy/negotiation skills			0.798^***^	1.69	0.98
16. Behaviors based on immediate, material and external objectives.			0.874^***^	1.80	1.07
17. Affected by traumatic experience			0.468^***^	0.94	1.11
18. Drug abuse			0.875^***^	1.32	1.16
19. Drug use interferes significantly with their lives/crimes related to drug use			0.860^***^	1.13	1.18
20. Friends with regular consumption			0.769^***^	1.90	1.09
21. Favorable attitude towards consumption			0.757^***^	1.38	1.06
**Socio-familial integration**	0.85	0.85			
1. Absence of rules and limits in the family system			0.754^***^	1.72	1.09
2. Contradictory, incoherent or inconsistent educational guidelines/rules			0.742^***^	1.75	1.13
3. Continuous negative communication and messages. Undervaluation of the child			0.515^***^	0.89	1.07
4. Difficulty or inability to control the child's behavior.			0.929^***^	2.02	1.07
5. Active resistance to educational guidelines/norms/rules			0.916^***^	1.50	1.15
6. Independent lifestyle, outside of family life			0.702^***^	1.38	1.18
7. Experience of family violence (direct or indirect)			0.488^***^	0.88	1.17
8. Reversal of parent/child roles			0.623^***^	0.95	1.11
**Social-interpersonal integration**	0.89	0.89		1.94	1.09
1. Antisocial relationship group			0.881^***^	0.93	1.16
2. Related to an organized violent group			0.500^***^	0.47	0.76
3. Lacks strategies to withstand peer pressure			0.551^***^		
4. Little or no presence of structured activities			0.940^***^	2.11	1.08
5. Leisure time is unoccupied and empty of content.			0.950^***^	2.13	1.05
6. Their personal interests and hobbies are maladaptive.			0.954^***^	1.70	1.18
7. Relates to and moves in marginal or highly conflictive social environments.			0.796^***^	1.62	1.14
8. Lack of social support			0.630^***^	1.02	1.07
9. Refuses intervention or does not know how to access social resources or sociocommunity resources.			0.806^***^	1.33	1.16

*** *p < 0.001*.

### Evidence of Validity

The Spearman correlation indexes between the PREVI-A dimensions and the YLS/CMI dimensions confirmed the existence of a significant association between all dimensions of both scales, with the exception of the correlation between the second dimension of PREVI-A (context-intervention) and dimension 1 of the YLS/CMI (criminal history). The estimated correlation indices ranged from *rs* = 0.47 (*p* < 0.001) to *rs* = 0.16 (*p* < 0.05), supporting the concurrent validity of the scale to measure the risk of delinquent behavior among juvenile offenders (Table 3).

**Table 3:** Hierarchical logistic regression model predicting criminal recidivism on PREVI-A dimensions.

**PREVI-A**	**β**	***SE***	**Wald**	**Exp(*B*)**	***CI***
Legal Situation	0.136^*^	0.065	4.404	1.146	1.009 1.301
Context and Intervention	−0.020	0.041	0.226	0.981	0.904 1.063
School, Occupational Training and Work	0.103^*^	0.050	4.227	1.108	1.005 1.223
Personal Development	0.028	0.027	1.072	1.028	0.976 1.083
Socio-Familial Integration	−0.003	0.044	0.004	0.997	0.915 1.087
Social-Interpersonal Integration	0.157^**^	0.050	10.058	1.170	1.062 1.289
Constant	−1.357	0.397	11.700	0.257	
*R^2^ Nagelkerke*					0.35
–*2 Log likehood*					196.17

^***^ *p < 0.001;*

** *p < 0.01;*

* *p < 0.05*.

In terms of predictive validity, the logistic regression model allowed us to correctly classify 78.6% of the minors on the risk of criminal recidivism and to explain 35% of the total variance. Higher scores in the Legal Situation (β = 0.136; *p* < 0.05), School, Occupational Training and Work (β = 0.103; *p* < 0.05), and Social/Interpersonal Integration (β = 0.157; *p* < 0.01) areas were significantly related to the risk of criminal recidivism (Table 3).

### PREVI-A Diagnostic Performance

**Figure 1:**
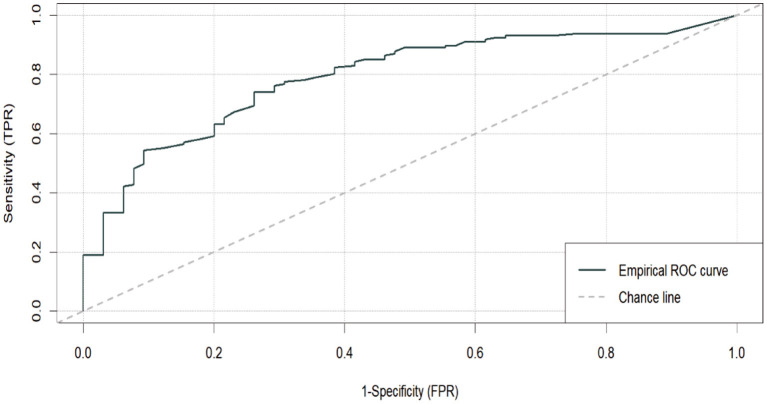
PREVI-A ROC curve on criminal recidivism.

Finally, the diagnostic performance of PREVI-A in discriminating the risk of recidivism vs. non-recidivism was estimated using a ROC curve (see Figure 1). The AUC (area under the curve) values calculated using the ROC test can range from 0.0, indicating no predictive validity, to 1.0, indicating perfect predictive validity. Values above 0.556 are considered low, 0.639 moderate and 0.714 high (Rice and Harris, 2005). The area under the curve (AUC) in PREVI-A was equal to 0.795, supporting high discriminative validity for the risk of criminal recidivism of juvenile offenders. Based on the estimated true positive, true negative and false positive rates, the most suitable cut-off point for predicting the risk of PREVI-A recidivism was determined to be 80. This cut-off point had an associated sensitivity (true recidivists) of 78%, and a false positive rate of 29%. Four PREVI-A risk levels were established based on these rates: Low = total scores of 0–39, Medium = total scores of 40–79, High = scores of or above 80, and Very High = total scores above 124. Finally, the diagnostic performance of each of the PREVI-A dimensions was tested. The results indicated that the dimensions of legal situation (AUC = 0.74), personal development area (AUC = 0.77), social-family integration (AUC = 0.73) and social-interpersonal integration (AUC = 0.80) had a high discriminative capacity, while the dimensions of the context and intervention (AUC = 0.70) and school-training-pre-work (AUC = 0.70) had a moderate discriminative capacity.

## Discussion

Our findings from this study empirically demonstrate the reliability and validity of PREVI-A for the assessment/evaluation of the risk of recidivism in relation to delinquent behavior by juvenile offenders, and support the factorial structure of the scale and its usefulness as a tool to obtain objective indicators for the educational and psychological aspects requiring treatment among the population of juvenile and adolescent offenders in Spain. The statistical analyses carried out further corroborated the theoretical factorial structure of the PREVI-A formed by six risk/protection dimensions (64 items) developed from the RNR model (Andrews and Bonta, 2010b), as well as the risk assessment requirements identified in the course of daily monitoring and treatment of juvenile offenders by ARRMI professionals over the last decade.

The alpha and omega internal consistency coefficients found evidence of good to excellent reliability for all six dimensions of PREVI-A (Legal Situation, Context and Intervention, School, Occupational Training and Work, Personal Development, Socio-Familial Integration, Social/Interpersonal Integration), while the indicators calculated using the Spearman correlation coefficient supported measurement stability after a period of 3 months. This evidence of validity reflects the precision and measurement stability of the PREVI-A, making it an ideal instrument to assess the risk of criminal recidivism and to explore the concurrence and dynamic interaction of risk/protection factors associated with the process of recidivism and desistance among young offenders (Barnes et al., 2016).

The evidence supporting the test-retest measurement stability of PREVI-A stands out as one of the strengths of the scale for risk assessment/evaluation, because initial risk assessment should not only respond to the need for classification of young offenders in terms of the risk of recidivism vs. non-recidivism according to the RNR model, but should also provide a route map for case-by-case management of each juvenile and a guide for the design of risk reduction strategies (Andrews et al., 2006). Ensuring the stability of measurement is critical if an instrument is be used to monitor change over time, and to assess and fine-tune therapeutic and intervention strategies (Vose et al., 2009). In this regard, it would be of interest for future research to explore the changes arising in the juvenile offenders' scores for each of the six dimensions of PREVI-A while they remain subject to correctional measures, insofar as the patterns and speed of change in the different measures would likely be more informative than a mere snapshot of the overall pre-post compliance change in the risk of subsequent recidivism (Cohen et al., 2016).

The evidence for construct validity reveals a significant relationship between the PREVI-A and the YLS/CMI criminal recidivism risk assessment instrument, supporting the concurrent validity of the risk assessment test. The Offenses and Measures dimension of the YLS/CMI and the Context and Intervention dimension of the PREVI-A were the only ones that did not correlate significantly. The explanation for this disconnect between the two measures may lie in the limited variability of the YLS/CMI resulting from the homogeneity of the study participants, all of whom were ordered into the ARRMI program by the courts, an effect that would in all probability be accentuated by the dichotomous response format of the YLS/CMI. However, future research should not rule out a more in-depth exploration of the relationship between these dimensions, and of possible interaction patterns in the scores offered by both in relation to recidivism by juvenile delinquents, since the lack of overlap could itself provide relevant information on recidivism patterns and the specific needs of the different samples according to the existing literature (Cohen et al., 2016).

Meanwhile, the PREVI-A displayed high predictive validity for the risk of criminal recidivism, with an AUC of 0.792, which is much higher than the mean AUC effects reported for two of the most widely used instruments in the United States (WSJCA and YLS/CMI), which range between 0.579 and 0.67 (Schmidt et al., 2005; Olver et al., 2009), and again for the YLS/CMI previously used in Spain, which showed an AUC equal to 0.71. This evidence of validity represents the second major strength of the test compared to pre-existing measurement instruments, which would position it as an optimal instrument for discriminating minors at risk of recidivism from those not at risk, as well as for follow-up and change in risk. It is recommended that future research prolong follow-up tracking of risk in juvenile offenders to corroborate the measurement stability of the PREVI-A in the Spanish context, given that recent research has reported changes in the AUC effect using follow-ups longer than 18 months (Andrews et al., 2011).

Despite the important contributions made by the present study to the assessment of the recidivism risk among juvenile offenders in Spain, a number of limitations exist which would affect interpretation of the results presented here. First and foremost, the results described in this paper may be skewed despite the adequacy of the sample size (*n* > 200 participants; Comrey and Lee, 1992) for the statistical analysis of the PREVI-A tool, by the unrepresentativeness of the study participants compared to the overall population of juvenile offenders in Spain. Second, the sample was drawn entirely from ARRMI centers, which could limit the variability of the data obtained. Finally, it was not possible given the sample size to corroborate the factorial invariance by gender or nationality of the juvenile offenders. Hence, findings with regard to the difference or similarity of the scores obtained from the application of the PREVI-A to boys and girls and across nationalities should be interpreted with caution until there is firm evidence of invariance validity by gender.

## Data Availability Statement

The datasets presented in this article are not readily available because “Data are available on reasonable request and on signature of a confidentiality agreement from author JG.” Requests to access the datasets should be directed to “JG, jlgranna@ucm.es”.

## Ethics Statement

The studies involving human participants were reviewed and approved by Deontological Commission of the Faculty of Psychology at the Complutense University of Madrid. The participants provided their written informed consent to participate in this study. Written informed consent to participate in this study was provided by the participants' legal guardian/next of kin.

## Author Contributions

All authors contributed to the conceptualization, investigation, formal analysis, and writing. All authors have read and agreed to publish the manuscript current version.

## Conflict of Interest

The authors declare that the research was conducted in the absence of any commercial or financial relationships that could be construed as a potential conflict of interest.

## Publisher's Note

All claims expressed in this article are solely those of the authors and do not necessarily represent those of their affiliated organizations, or those of the publisher, the editors and the reviewers. Any product that may be evaluated in this article, or claim that may be made by its manufacturer, is not guaranteed or endorsed by the publisher.
